# Revealing factors influencing polymer degradation with rank-based machine learning

**DOI:** 10.1016/j.patter.2023.100846

**Published:** 2023-09-25

**Authors:** Weilin Yuan, Yusuke Hibi, Ryo Tamura, Masato Sumita, Yasuyuki Nakamura, Masanobu Naito, Koji Tsuda

**Affiliations:** 1 Graduate School of Frontier Sciences, The University of Tokyo, 5-1-5 Kashiwanoha, Kashiwa, Chiba 277-8561, Japan; 2 Research Center for Macromolecules and Biomaterials, National Institute for Materials Science, 1-2-1 Sengen, Tsukuba, Ibaraki 305-0047, Japan; 3 Center for Basic Research on Materials, National Institute for Materials Science, 1-1 Namiki, Tsukuba, Ibaraki 305-0044, Japan; 4 RIKEN Center for Advanced Intelligence Project, 1-4-1 Nihonbashi, Chuo-ku, Tokyo 103-0027, Japan

**Keywords:** polymer degradability prediction, rank-based machine learning, RankSVM, exposure experiments, marine sustainability, degradability factors analyzation, data integration

## Abstract

The efficient treatment of polymer waste is a major challenge for marine sustainability. It is useful to reveal the factors that dominate the degradability of polymer materials for developing polymer materials in the future. The small number of available datasets on degradability and the diversity of their experimental means and conditions hinder large-scale analysis. In this study, we have developed a platform for evaluating the degradability of polymers that is suitable for such data, using a rank-based machine learning technique based on RankSVM. We then made a ranking model to evaluate the degradability of polymers, integrating three datasets on the degradability of polymers that are measured by different means and conditions. Analysis of this ranking model with a decision tree revealed factors that dominate the degradability of polymers.

## Highlights

• Existing datasets of polymer degradability are small and often incompatible
• A rank-based method for integrating diverse polymer degradability datasets is presented
• Key molecular factors that determine degradability are identified

## The bigger picture

In view of marine environmental protection, degradation control of polymers is an increasingly important topic. Existing datasets about polymer degradability are small and diverse in measurement conditions, which makes direct comparison across datasets notoriously difficult. Improved machine learning methods that better leverage limited and incompatible data, such as the RankSVM-based method described in this work, may help chemists develop new and better polymers.

## Graphical abstract

**Figure:**
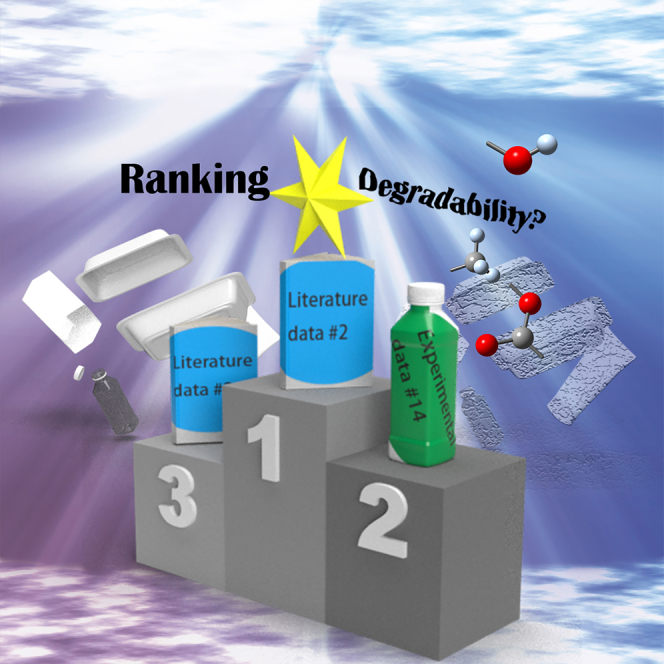

## Teaser

Factors influencing polymer degradability are identified via machine learning-based integrated analysis. This approach opens a way to large-scale analysis of polymer degradability, which has been hampered by the extremely diverse measurement conditions of existing datasets.

## Introduction

In view of marine environmental protection, degradation control of polymers is an increasingly important topic.^1^^,^^2^ Plastic waste is a major problem because it takes time to degrade in the ocean.^3^ Over a long time, plastic waste is gradually degraded to smaller sizes by waves, wind, and some environmental factors that contribute to the molecular level.^4^ In particular, microplastics smaller than 5 mm are known to cause a significant impact on the ecosystem and are a major social problem.^5^ Therefore, the development of polymers with controllable degradability is important for protecting the marine environment.

To develop polymers with controllable degradability, it is necessary to consider the degradation at the chemical level. From the chemical viewpoint, three important degradation mechanisms are known.^4^ The first is photo-degradation by ultraviolet (UV) light. Impurities, heterostructures, or the polymer itself are photodegraded, leading to a decrease in molecular weight and cracking.^6^ The second is hydrolysis in which polymers are degraded by water. It is known that the higher temperature causes a shorter degradation time.^6^ The third is biodegradation. Bacteria attach to the surface of polymers and form biofilms, which accelerate degradation.^7^ In the ocean, a complex interplay of these factors leads to the degradation of polymers. Therefore, it is difficult to conduct experiments in which these factors are uniquely controlled, and many of the data reported on degradability were highly dependent on environments.

In recent years, machine learning has been used to analyze polymer data to extract factors that contribute to physical properties.^8^ However, applying machine learning to analyze the degradability does not facilitate the analysis because the strong environmental dependence of the data inhibits the direct comparison of them. To circumvent this obstacle, we used ranking-based machine learning^9^^,^^10^ to integrate datasets measured in different environments and have built a unified ranking of degradability through RankSVM,^11^^,^^12^ which can learn the pairwise preference. After training RankSVM, degradability scores of all polymers have been obtained. Based on this score, we have analyzed the important factors that dominate the degradability of polymers with tree-based analysis. Furthermore, using the constructed RankSVM model, we have predicted the ranking of a polymer database (4,577 polymers) where the values of the degradability of polymers are not measured.

## Results and discussion

### Degradation datasets

To demonstrate the effectiveness of our method, we made a dataset consisting of 15 polymers we measured (experimental datasets 1 and 2), and 24 polymers were obtained from the literature,^13^ as the literature dataset, summarized in Table 1. Surely, the former and latter datasets are measured in different conditions. For preparing our dataset, seven polymer films were produced by drying the homopolymer solutions on a glass slide as experimental dataset 1. Each polymer film is placed in a centrifuge tube and utterly immersed in artificial seawater. The samples were placed on the roof of the National Institute for Materials Science (Tsukuba, Japan) and applied to the exposure experiments. The results of degradation in experimental dataset 1 are summarized in Table 2. Polymer degradability was measured via the concentration of total organic carbon (TOC) in the artificial seawater after the exposure experiment. In detail, the degradability δ was evaluated by Equation 6 from the weight of the polymers $W_{\text{film}}$, the surface area of the polymers $S_{\text{film}}$, the volume of seawater $V_{\text{water}}$, and the proportion of carbon elements $M_{c}$%. For the other dataset, eight commercial homopolymer film products with uniform thickness were prepared as experimental dataset 2, which are summarized in Table 3. For experimental datasets 1 and 2, since the exposure experiments were conducted under natural conditions, UV exposure and temperature changes are the main factors for the degradation.

**Table 1:** Summary of literature dataset from Min et al., in which the ranking was created by weight loss per day

Name	Abbreviation	SMILES	Wt. loss (%/day)
Poly(ethylene terephthalate)^14^	PET	∗CCOC(=O)c1ccc(*cc*1)C(=O)O∗	0.0003
Poly(lactic acid)^15^	PLA	∗OC(C)C(=O)∗	0.00033
Poly(L-lactic acid)^16^	PLLA	∗C([C@H](O∗)C) = O	0.0014
Polycaprolactone^14^	PCL	∗CCCCCC(=O)O∗	0.0027
Polystyrene^17^	PS	∗CC(∗)c1ccccc1	0.0111
Polyurethane^18^	PU	∗C(=O)NC1 = CC = C(CC2 = CC = C(NC(=O)OCCO∗)C=C2)C=C1	0.0116
Nylon6^19^	Nylon6	∗NCCCCCC(∗) = O	0.0222
Polycarbonate^20^^,^^21^	PC	∗Oc1ccc(C(C)(C)c2ccc(OC(∗) = O)cc2)cc1	0.0238
Poly(butylene succinate)^22^	PBS	∗OCCCCOC(=O)CCC(∗) = O	0.0714
Nylon66^19^	Nylon66	∗NCCCCCCNC(=O)CCCCC(∗) = O	0.0778
Poly(ethylene sebacate)^23^	PESeb	∗OCCOC(=O)CCCCCCCCC(∗) = O	0.0894
Poly(butylene sebacate)^23^	PBSeb	∗OCCCCOC(=O)CCCCCCCCC(∗) = O	0.114
Poly(butylene azelate)^23^	PBAz	∗OCCCCOC(=O)CCCCCCCC(∗) = O	0.2011
Poly(3-hydroxybutyrate)^24^	P3HB	CC(CC(∗) = O)O∗	0.2917
Poly(ethylene azelate)^23^	PEAz	∗OCCOC(=O)CCCCCCCC(∗) = O	0.3073
Poly(butylene adipate)^22^	PBAdip	∗OCCCCOC(=O)CCCCC(∗) = O	0.3929
Nylon4^25^	Nylon4	∗NCCCC(∗) = O	1.4286
Poly(propylene succinate)^23^	PPS	∗OCCCOC(=O)CCCCCCC(=O)∗	2.2405
Poly(vinyl alcohol)^26^	PVA	C(C(O)∗)∗	2.8333
Poly(propylene azelate)^23^	PPAz	∗OCCCOC(=O)CCCCCCCC(=O)∗	7.4969
Poly(propylene sebacate)^23^	PPSeb	∗OCCCOC(=O)CCCCCCCCC(∗) = O	7.5642
Poly(propylene adipate)^27^	PPAd	∗OCCCOC(=O)CCCCC(=O)∗	61.3197
Poly(propylene glutarate)^27^	PPGI	∗OCCCOC(=O)CCCC(=O)∗	100
Poly(propylene pimalate)^28^	PPPIM	∗OCCCOC(=O)CCCCCC(∗) = O	100

**Table 2:** Results of degradation in experimental dataset 1

Name	Abbreviation	SMILES	W_film_ [mg]	TOC [mg/L]	V_water_ [mL]	M_c_ [%]	S_film_ [cm^2^]	δ
Poly(isopropyl acrylate)	PIPA	∗CC(∗)C(=O)OC(C)C	34.1	0.5705	62	63.16	7.48	0.0002196
Poly(isodecyl acrylate)	PIDA	∗CC(∗)C(=O)OCCCCCCCC(C)C	16.1	0.5347	57	73.58	8.46	0.0003041
Poly(benzyl acrylate)	PBA	∗CC(∗)C(=O)OCc1ccccc1	19.4	0.749	47	74.07	6.63	0.0003695
Poly(2-methoxyethyl acrylate)	PMEA	∗CC(∗)C(=O)OCCOC	12.6	0.5426	68	55.38	12.60	0.0004196
Poly(2-butoxyethyl acrylate)	PBEA	∗CC(∗)C(=O)OCCOCCCC	9.9	0.7745	50	62.79	14.00	0.0004450
Poly(vinyl butyral)	PVB	∗CC1CC(∗)OC(CCC)O1	11	0.6276	50	67.61	7.20	0.0005861
Poly(hexamethylene sebacate)	PHMS	∗CCCCCCOC(=O)CCCCCCCCC(=O)O∗	8.8	0.4898	76	67.61	9.69	0.0006457

**Table 3:** Results of degradation in experimental dataset 2

Name	Abbreviation	SMILES	W_film_ [mg]	TOC [mg/L]	V_water_ [mL]	M_c_ [%]	S_film_ [cm^2^]	δ
Polystyrene	PS	∗CC(∗)c1ccccc1	1.703	0.9217	32	0.05778	8.8	0.006566
Polypropylene	PP	∗CC(∗)C	0.9463	0.8555	30	0.12047	6	0.02008
Polyacetal	POM	∗CO∗	1.071	0.3997	33	0.21463	10.08	0.02129
Poly(tetrafluoroethylene)	PTFE	∗C(∗)(F)F	0.9634	0.2400	39	0.21141	7.6	0.02782
Polycarbonate	PC	∗Oc1ccc(C(C)(C)c2ccc(OC(∗) = O)cc2)cc1	1.804	0.7551	36	0.19936	7.02	0.0284
Poly(methyl methacrylate)	PMMA	∗CC(∗)(C)C(=O)OC	1.128	0.5993	29	0.1631	3.8	0.04292
Poly(ethylene naphthalate)	PEN	∗CCOC(=O)c1ccc2cc(C(=O)O∗)ccc2c1	1.565	0.6936	25	0.48537	2.31	0.2101
Polychloroprene	CR	∗CC/C=C(/∗)Cl	6.878	0.5421	37	4.49634	9.66	0.4655

### Unified ranking integrating three datasets

The RankSVM model to predict degradability score was trained by using the pairwise preferences prepared from each dataset. Note that preferences cannot be obtained between different datasets. In the RankSVM, there is a hyperparameter C. The value of C is determined so that the prediction accuracy for preferences, which is evaluated by cross-validation, is maximized. Here, 5-fold cross-validation is performed, and the accuracy depending on C is shown in Figure S1. When C=0.007, the accuracy reached a maximum value of 0.85, confirming the high prediction accuracy for the preferences. The RankSVM model was trained using all preferences when C=0.007, and the unified ranking was created by arranging the polymers in order of their degradability scores d, which are calculated by Equation 5.

**Figure 1:**
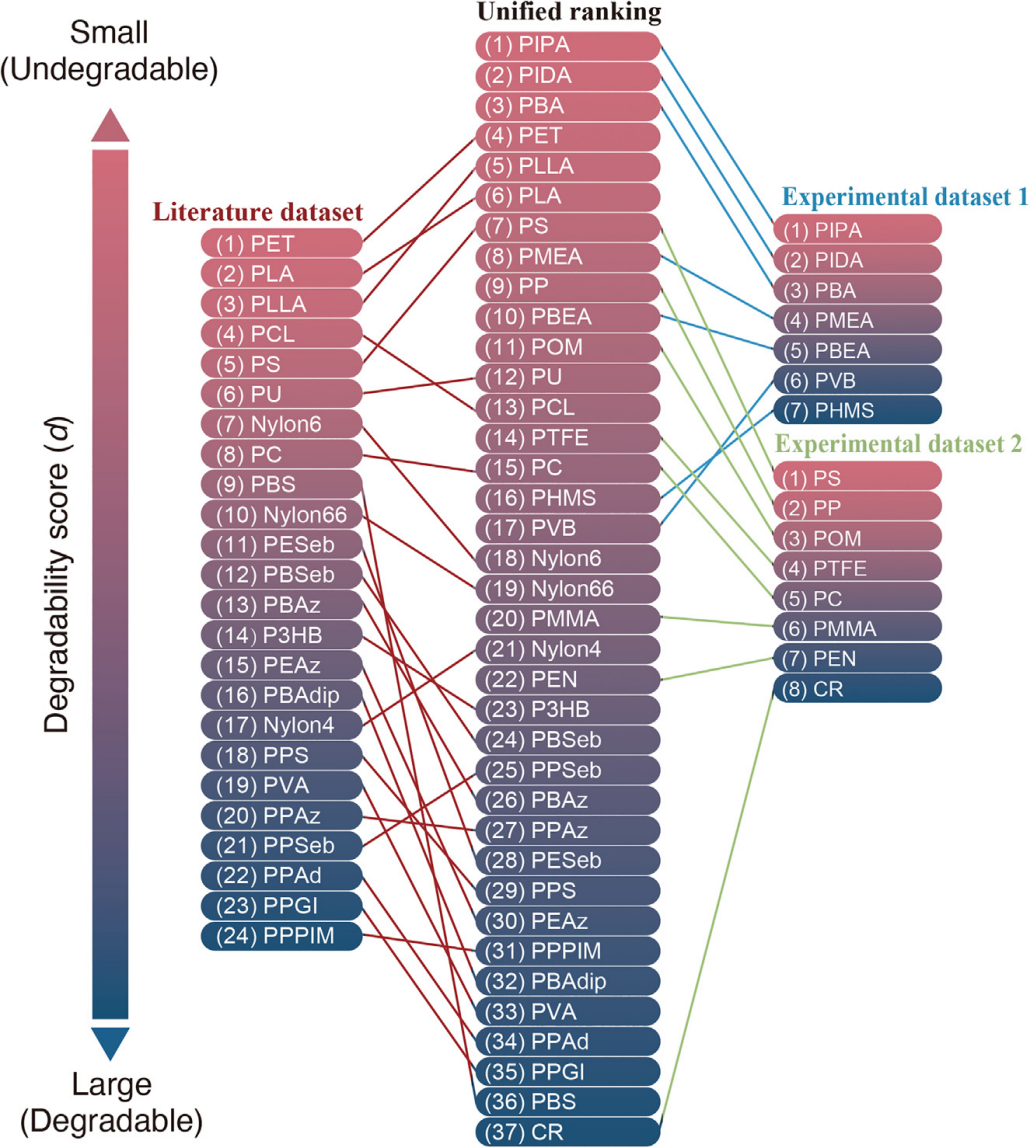
Rankings of polymer degradability The degradability scores in the unified ranking are derived from the RankSVM trained with the integration of the literature dataset and two experimental datasets. In the literature dataset, 24 data are contained from Min et al.^13^ For experimental datasets 1 and 2, seven polymer films were produced, and eight commercial homopolymers were prepared, respectively. The common polymers of the literature dataset and experimental dataset 2 are PS and PC.

The unified ranking made by RankSVM and individual rankings of each dataset are shown in Figure 1. It is noteworthy that the rankings are consistent, even though they are prepared via different measurement conditions and experiments. Experimental dataset 2 contains many acrylate polymers, which are regarded as undegradable polymers in the unified ranking. The polymers in experimental dataset 2 are scattered over the whole range of the unified ranking. PS and PC, the common polymers of the literature dataset and experimental dataset 2, lie at the reasonable ranking in the unified ranking. In other words, PC is more degradable than PS. This fact indicates that the unification of different datasets has succeeded, and the reliability of unified ranking is expected to be elevated by adding the datasets.

### Revealing factors influencing polymer degradation

**Figure 2:**
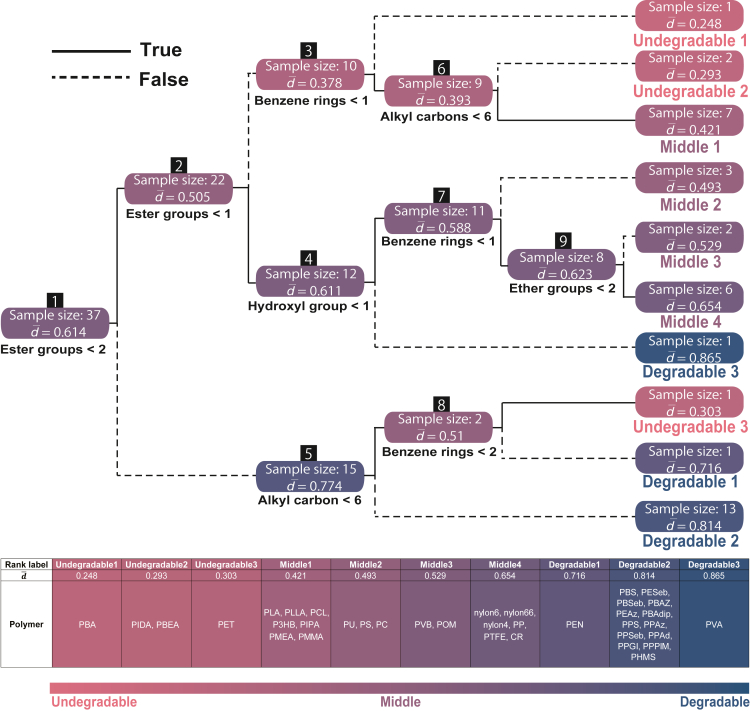
Regression tree trained using a degradability score derived from the RankSVM model Nine molecular properties of each polymer were used as descriptors, and five key properties were selected for regression tree construction. The polymers were classified into three categories according to average degradability score $\bar{d}$ ($\bar{d}\leq 0.303$: “undegradable,” $0.421\leq \bar{d}\leq 0.654$: “middle,” and $\bar{d}\geq 0.716$: “degradable”).

To extract the factors that dominate the degradability of a polymer, we made a regression tree, as shown in Figure 2, with nine molecular properties as input and the degradability score d as output. Here, we used molecular properties, including information on chemical composition, which are familiar factors for synthetic chemists. According to this tree, the numbers of ester groups, alkyl carbons, hydroxyl groups, ether groups, and benzene rings are selected as key descriptors. On the other hand, the numbers of the amide group, carbonates, hetero atoms, and urethane are not selected as key descriptors. In this tree, the number of leaf nodes is 10, and they are classified into three categories, “undegradable” ($\bar{d}\leq 0.303$), “middle” ($0.421\leq \bar{d}\leq 0.654$), and “degradable” ($\bar{d}\geq 0.716$), according to the average degradability scores of polymers in each category, denoted by $\bar{d}$.

The prediction result of the decision tree mainly shows the following tendency. (1) The polymers with more ester groups tend to be more degradable (node 1). (2) The polymers with benzene rings tend to have low degradability (nodes 3 and 7). (3) The polymers with more alkyl carbons tend to be more undegradable (node 6). Concerning (1), the polymers with ester groups are known to be susceptible to alkaline environments of marine and microbial action.^28^ The decision tree successfully captured these characteristics. Our analysis with the decision tree also reproduces the characteristics. For (3), in general, more alkyl carbons in monomer structures are known to promote crystallization, which tends to elevate the glass transition temperature ($T_{g}$).^29^ Higher crystallinity and $T_{g}$ would be favorable for reducing degradability.^30^ On the other hand, node 5 shows the opposite trend to node 6. The branching at node 5 can be considered as the presence or absence of benzene rings, which happens to match the factor for a number of alkyl carbons. Then, node 5 extracts the factor that the polymers without benzene rings are more degradable, which is consistent with the fact extracted as (2).

Contrary to factor (2), node 8 is a branch for classifying the polymers with more benzene rings to degradable ones (like PET [benzene ring = 1] and PEN [benzene ring = 2]). PEN has two benzene rings, but these form a naphthalene ring. Thus, the degradation mechanism of PEN would be different from that by polymers with some independent benzene rings such as PU and PC, and node 8 is not directly related to the mechanism of (2). Let us consider the difference between the degradation mechanisms of PET and PEN. First, since PEN has higher $T_{g}$ than PET, PEN would have higher thermal stability.^31^ On the other hand, the higher $T_{g}$ causes degradation in the PEN film during high-temperature processing,^32^ and it is difficult to compare them from their values of $T_{g}$. Second, PEN is susceptible to photo-oxidative degradation under UV light conditions, which causes yellowing and gel formation on the surface. In addition, it is known that PET exhibits photo-stability higher than PEN.^33^ Therefore, in terms of photo-degradation, PET would be ranked as more undegradable than PEN in the unified ranking. Note that these data come from different incompatible datasets, and this node is extracted by integrating some datasets.

Interestingly, the decision tree shows that PVA categorized in degradable 3 is an exceptional degradable polymer despite the absence of ester groups. We speculate that the impurity in PVA largely affects the degradability of PVA. PVA is usually synthesized through hydrolysis of polyvinyl acetate resulting in impurities in PVA.^34^ Hence, the sample should include some impurities, and measuring the accurate degradability of pure PVA is difficult. The decision tree also classified PET in undegradable 3 as an undegradable polymer in spite of its ester groups. We guess that the stabilization effect of the π-stacked structure can significantly reduce the degradability even if the monomer structure contains two ester groups. On the other hand, it is also known that the degradability of PET increases with increasing the fraction of the ester group.^35^

### Predictions of degradable or undegradable polymers in PoLyInfo

The degradability scores of polymers recorded in PoLyInfo^36^ are predicted by using our model. We obtained 17,771 SMILES data from PoLyInfo, and 4,577 polymers were extracted by considering the applicability domain. Here, the applicability domain is determined by a k-nearest neighbors-based approach method.^37^ The top 10 undegradable and degradable polymers as prediction results are shown in Tables 4 and 5. It can be found that the model tends to predict that the acrylate polymers and polymers with an amide group are more undegradable. The polymers with an amide group show certain stability in a water environment and usually have high $T_{g}$ due to the hydrogen bonds effect.^38^^,^^39^ On the other hand, the predicted degradability is widely distributed, even if they have the same functional group (see Figure S2), since it is not predicted only from certain functional groups. Therefore, the polymers shown in Tables 4 and 5 are ranked high not only by functional groups but also by other factors such as number of atoms and types of neighbor atoms.

**Table 4:** Prediction results of top 10 undegradable polymers in PoLyInfo, where the name of the polymer, degradability score *d* obtained by RankSVM, and chemical structure are shown

Name	Degradability score	Chemical structure
Poly[1-(1-{[1-(ethoxycarbonyl)ethoxy]carbonyl}ethyl)pyrrole-2,5-diyl]	−0.296575962	
Poly(N,N-diisopropylacrylamide)	−0.210303998	
Poly(isopropyl crotonate)	−0.108607135	
Poly{bis[(4-(methoxycarbonyl)phenoxy]phosphazene}	−0.052889856	
Poly(diisopropyl fumarate)	−0.024667207	
Poly[4-(isopropoxycarbonyl)styrene]	−0.020469478	
Poly(4-isopropoxystyrene)	−0.01198865	
Poly(isopropyl acrylate)	0.00000003	
Poly{1-[(dioctylamino)carbonyl]ethylene}	0.033141513	
Poly{(butane-1,4-diamine)-alt-[4,4'-(propane-1,2-diyldioxy)dibenzoic acid]}	0.036542042

**Table 5:** Prediction results of top 10 degradable polymers in PoLyInfo, where the name of the polymer, degradability score *d* obtained by RankSVM, and chemical structure are shown

Name	Degradability score	Chemical structure
Poly{1-[3-(4-benzoyl-3-hydroxyphenoxy)-2-hydroxypropoxycarbonyl]-1-methylethylene}	1.971466427	
Poly[3,3-bis(hydroxymethyl)oxetane]	1.822278633	
Poly{[4,5-bis(hydroxymethyl)cyclopentane-1,3-diyl]ethene-1,2-diyl}	1.822115257	
Poly(glyceryl methacrylate)	1.817512141	
Poly[iminoglutarylimino(3,3′-dihydroxybiphenyl-4,4′-diyl)]	1.780371805	
Poly(1,4-dihydroxy-1-methylbutane-1,4-diyl)	1.637276638	
Poly(3-chloro-2-hydroxypropyl methacrylate)	1.611935588	
Poly{[2,2-dimethyl-4,5-bis(hydoxymethyl)-1,3-dioxolane]-alt-(diethyl carbonate)}	1.603114876	
Poly{1-[2-(5-bromobenzofuran-2-yl)-2-oxoethoxycarbonyl]-1-methylethylene}	1.559941678	
Poly{2-hydroxy-5-[1-(4-hydroxyphenyl)-1-methylethyl]-1,3-phenylene}	1.548126408	

The top 10 degradable polymers almost all contain a hydroxyl group. On the other hand, the position of a hydroxyl group largely influences the polymer degradability, and the mechanism should be different between the degradation of the side chain via hydrolysis and that of the polymer backbone. The position of a hydroxyl group largely influences the polymer degradability. However, it is currently difficult to distinguish between these degradation mechanisms because the training data do not contain sufficient data. Since SMILES contains information on the polymerization point, the Mol2vec descriptor can distinguish whether hydroxyl groups are on the side chains or on the polymer backbone. Thus, we will be able to distinguish it by conducting experiments on the polymers shown in Table 5. In addition, in the training data, there are no polymers with multiple hydroxyl groups, and the predicted degradability for such polymers would be unreliable. To avoid various limitations for prediction, using these prediction results, it is necessary to increase the amount of experimental data.

### Conclusions

**Figure 3:**
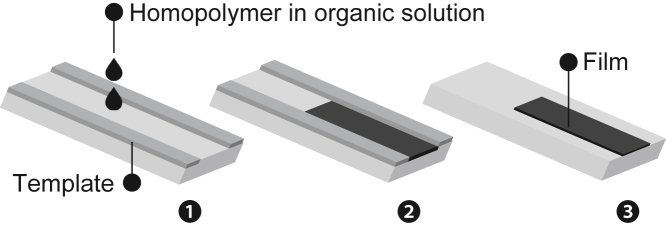
Schematic pictures for the preparation process of polymer films in experimental dataset 1 (1) Polymer solution is attached to the glass slide. (2) Drying is performed to obtain polymer films. (3) The template is removed.

In conclusion, we have presented a platform that can evaluate polymer degradability based on incompatible datasets through rank-based machine learning. In this study, three types of degradation datasets have been prepared. See Tables 6 and 7 and Figure 3 for experimental details. For each dataset, we have evaluated preferences, and the RankSVM model has been trained to predict preferences. Based on the degradability score obtained by a trained RankSVM model, a unified ranking has been constructed. To reveal effective factors for the polymer degradation, the decision tree has been trained. The characteristic of this decision tree is that the input is molecular properties, and the output is the degradability score, which is evaluated by integrating incompatible datasets. For input molecular properties, we have used chemical compositions such as the numbers of ester groups, benzene rings, and alkyl carbons, which are familiar to synthetic chemists. From the decision tree, we conclude that the learning of rank-based models works well because the well-known factors in polymer science are extracted. On the other hand, there are no restrictions on the properties as input for the decision tree, and morphology descriptors such as distance between rings and length of the main chain will be target factors that are easy to understand. Although there is still ambiguity and lack of knowledge on the factors of monomer structure that influence the degradability of polymers, the prediction results are considerably consistent with existing knowledge. Because a higher accurate unified ranking helps to deepen the understanding of the polymer degradability, further experiments will be applied to increase the data and improve the accuracy of the prediction model as future prospects.

As discussed in Jablonka et al.,^40^ large-scale collection of chemical data has the potential to change the way of material discovery. The unified ranking we have shown in this study is still small, but technically, it is possible to construct a ranking for millions of polymers. In the future, our data integration approach will be improved by a growing amount of collective data and will grow into an important resource that chemists can easily refer to and use for developing polymers.

## Experimental procedures

### Resource availability

#### Lead contact

Further information and requests should be directed to and will be fulfilled by the lead contact, Koji Tsuda (tsuda@k.u-tokyo.ac.jp).

#### Materials availability

This study did not generate any new unique materials.

### Calculation details in RankSVM

In RankSVM, each dataset is converted to pairwise preferences by using molecular fingerprints of polymer 1 ($x_{i}^{\left(1\right)}$) and polymer 2 ($x_{i}^{\left(2\right)}$) for comparison and summarized into one training set,

(Equation 1) $$D={\left\{\left(x_{i}^{\left(1\right)},x_{i}^{\left(2\right)}\right),t_{i}\right\}}_{i=1}^{M}\text{,}$$

$$t_{i}=\left\{ \begin{array}{c} 1,\text{if}\;\text{the}\;\text{degradability}\;\text{of}\;\text{polymer}\;1\;\text{is}\;\text{larger}\;\text{than}\;\text{polymer}\;2\\ -1,\text{otherwise} \end{array}\right.\text{,}$$

where M is the number of total preferences. We employed 300-dimensional Mol2vec as the molecular fingerprint.^41^ RankSVM uses the following prediction function:

(Equation 2) $$y=w^{T}\left(x_{i}^{\left(1\right)}-x_{i}^{\left(2\right)}\right)\text{.}$$

The parameter w is estimated by solving the following optimization problem such that the predicted results are maximally consistent with the labels $t_{i}$:

(Equation 3) $$\left[w^{*},\xi^{*}\right]=\arg\min_{w,\xi }\left\|w\right\|+C\sum_{i=1}^{M}\xi_{i}\text{,}$$

(Equation 4) $$t_{i}\left[w^{T}\left(x_{i}^{\left(1\right)}-x_{i}^{\left(2\right)}\right)\right]\geq 1-\xi_{i}\;\left(i=1,\ldots ,M\right)\text{,}$$

where $\xi_{i}$ is a slack parameter, and C is a trade-off parameter as hyperparameter. After training RankSVM, we can compute the degradability score d as the following for any polymer:

(Equation 5) $$d={\left(w^{*}\right)}^{T}x$$

Through Equation 5, any polymer from the training data or from other databases such as PolyInfo^36^ can be included in the unified ranking if only the molecular fingerprint can be generated.

In our implementation, Python script degradability_ranking.py integrates the degradability datasets and outputs the unified ranking shown in Figure 1. The input files for this script are literature.xlsx, exp1.xlsx, and exp2.xlsx, each of which contains the SMILES strings of polymers and their degradability values. The rankings shown in Tables 4 and 5 were obtained using main.py.

### Materials

The raw materials for polymer film in experimental dataset 1 are summarized in Table 6. The commercially available polymer films in experimental dataset 2 are summarized in Table 7. The polymer films were cut into small pieces and washed with deionized water. The artificial seawater was prepared by dissolving 18 g commercial powder of artificial seawater (Tomita Pharmaceutical Co., Tokushima, Japan) into 500 g of deionized water.

**Table 6:** Production companies, product number, composition, and shape of raw materials used in experimental dataset 1

Name	Company	Product number	Composition	Shape
PIPA	Scientific Polymer Products Inc., New York, USA	#475	poly(isopropyl acrylate): 25%–30%, toluene: 70–75%	liquid
PIDA	Scientific Polymer Products Inc., New York, USA	#875	poly(isodecyl acrylate):25%–30%, toluene: 70%–75%	liquid
PBA	Scientific Polymer Products Inc., New York, USA	#883	poly(benzyl acrylate): 30%–35%, toluene: 65–70%	liquid
PMEA	Scientific Polymer Products Inc., New York, USA	#891	poly(2-methoxyethyl acrylate): 20%–25%, toluene: 75%–80%	liquid
PBEA	Scientific Polymer Products Inc., New York, USA	#896	poly(2-butoxyethyl acrylate): 18%–22%, toluene: 78%–82%	liquid
PVB	Scientific Polymer Products Inc., New York, USA	#043	poly(vinyl butyral): 96.0%, 1,1-diethoxybutane: ≤2.0%, water: ≤2.0%	powder

**Table 7:** Production companies and product number of polymers in experimental dataset 2

Name	Company	Product number
PS	Hikari Co., Ltd., Osaka, Japan	PS2031-1
PP	Artec Co., Ltd., Osaka, Japan	20511
POM	ESCO Co., Ltd., Osaka, Japan	EA441MD-0.3
PTFE	Chukoh Chemical Industries, Ltd., Tokyo, Japan	ASF-110 FR
PC	Hikari Co., Ltd., Osaka, Japan	KPAC2005-1
PMMA	Asahi Kasei Co., Ltd., Tokyo, Japan	K120913
PEN	Teijin DuPont Films Japan Limited., Co., Ltd., Tokyo, Japan	Q51-A4
CR	P.D.R. Co., Ltd., Aichi, Japan	crop bara

### Film preparations in experimental dataset 1

The procedure of film preparation for the polymer powders is as follows: PVB and PHMS are polymer powders. The polymer powder 100 mg was dissolved in toluene 900 mg (Wako Pure Chemical Industries, Osaka, Japan). The resulting 10 wt % solutions were cast on the glass slide, which was in advance templated with Teflon tape to refine the film area (see Figure 3). After drying the casted solution in the air for an hour, the Teflon tape was removed from the glass slide. The polymer film was further dried in the vacuum oven at 100°C to completely remove the solvents. The PIPA, PIDA, PBA, PMEA, and PBEA are the samples as toluene solutions. For these samples, the same procedures with polymer powders were applied for film preparation.

### Degradation experiments

In an open-top glass container, the prepared films and commercially available polymer films were completely immersed in the artificial seawater. The top of the container was sealed with thin plastic wrap. The container was left on the roof of the National Institute for Materials Science (Tsukuba, Japan) for around 20–30 days (experimental dataset 1: 7/30/2021–8/19/2021 and experimental dataset 2: 11/5/2021–12/2/2021). After the degradation, the film was removed from the seawater. After vigorous mixing, 2 mL of the seawater was extracted and diluted 10 times with deionized water. For this liquid sample, TOC was measured.

### Total organic carbon measurement

The TOC measurements were conducted on TOC-L (Shimadzu). The calibration was conducted using sodium carbonate and potassium hydrogen phthalate aqueous solutions (100 C/L). The measurements were performed three times. For determining the absolute values of organic compounds dissolved/scattered in the seawater, we used the averaged TOC values to evaluate polymer degradability. The TOC was obtained by the difference between total carbon value (TC) and inorganic carbon value (IC), that is, TOC = TC – IC.

The weight $W_{\text{film}}$ and surface area $S_{\text{film}}$ of the polymers and the volume of artificial seawater $V_{\text{water}}$ were measured before degradation experiments. The weight of carbon dissolved in water was obtained by multiplying $V_{\text{water}}$ and TOC. The total weight of carbon elements in the film can be evaluated by multiplying the proportion of carbon elements in molecular weight $M_{c}$% and $W_{\text{film}}$. The density of polymers is assumed as 1 g/cm^3^. Considering the influence of surface area $S_{\text{film}}$, the degradability δ is defined as follows:

(Equation 6) $$\delta =\frac{\text{TOC}\times V_{\text{water}}}{W_{\text{film}}\times M_{c}\times S_{\text{film}}}\text{.}$$

The results of degradability for experimental dataset 1 and experimental dataset 2 are shown in Tables 2 and 3, respectively.

## Data Availability

All original code has been deposited at Zenodo under the https://doi.org/10.5281/zenodo.8268022 and is publicly available as of the date of publication. All polymer degradability datasets used in this paper and the predicted degradability scores of PoLyInfo polymers are deposited at Zenodo under the https://doi.org/10.5281/zenodo.8268022 and are publicly available as of the date of publication.

## Supplemental information

Document S1. Figures S1 and S2

Document S2. Article plus supplemental information

## References

[1] Vikhareva I.N., Buylova E.A., Yarmuhametova G.U., Aminova G.K., Mazitova A.K. (2021). An Overview of the Main Trends in the Creation of Biodegradable Polymer Materials. J. Chem..

[2] Kuenneth C., Lalonde J., Marrone B.L., Iverson C.N., Ramprasad R., Pilania G. (2022). Bioplastic design using multitask deep neural networks. Commun. Mater..

[3] Chamas A., Moon H., Zheng J., Qiu Y., Tabassum T., Jang J.H., Abu-Omar M., Scott S.L., Suh S. (2020). Degradation Rates of Plastics in the Environment. ACS Sustain. Chem. Eng..

[4] Pandey J.K., Raghunatha Reddy K., Pratheep Kumar A., Singh R.P. (2005). An overview on the degradability of polymer nanocomposites. Polym. Degrad. Stabil..

[5] Ivar do Sul J.A., Costa M.F. (2014). The present and future of microplastic pollution in the marine environment. Environ. Pollut..

[6] Lu T., Solis-Ramos E., Yi Y., Kumosa M. (2018). UV degradation model for polymers and polymer matrix composites. Polym. Degrad. Stabil..

[7] Ganesh Kumar A., Anjana K., Hinduja M., Sujitha K., Dharani G. (2020). Review on plastic wastes in marine environment – Biodegradation and biotechnological solutions. Mar. Pollut. Bull..

[8] Rasulev B., Casanola-Martin G. (2020). QSAR/QSPR in Polymers: Recent Developments in Property Modeling. Int. J. Quant. Struct. Prop. Relatsh..

[9] Sun X., Hou Z., Sumita M., Ishihara S., Tamura R., Tsuda K. (2020). Data integration for accelerated materials design via preference learning. New J. Phys..

[10] Sun X., Tamura R., Sumita M., Mori K., Terayama K., Tsuda K. (2022). Integrating Incompatible Assay Data Sets with Deep Preference Learning. ACS Med. Chem. Lett..

[11] Herbrich R., Graepel T., Obermayer K., Smola A.J., Bartlett P., Schölkopf B., Schuurmans D. (2000). Advances in large margin classifiers.

[12] Joachims T. (2002). Proceedings of the eighth ACM SIGKDD international conference on Knowledge discovery and data mining KDD ’02.

[13] Min K., Cuiffi J.D., Mathers R.T. (2020). Ranking environmental degradation trends of plastic marine debris based on physical properties and molecular structure. Nat. Commun..

[14] Bagheri A.R., Laforsch C., Greiner A., Agarwal S. (2017). Fate of So-Called Biodegradable Polymers in Seawater and Freshwater. Glob. Chall..

[15] Martin R.T., Camargo L.P., Miller S.A. (2014). Marine-degradable polylactic acid. Green Chem..

[16] Tsuji H., Suzuyoshi K. (2002). Environmental degradation of biodegradable polyesters 1. Poly(ϵ-caprolactone), poly[(R)-3-hydroxybutyrate], and poly(L-lactide) films in controlled static seawater. Polym. Degrad. Stabil..

[17] Syranidou E., Karkanorachaki K., Amorotti F., Avgeropoulos A., Kolvenbach B., Zhou N.-Y., Fava F., Corvini P.F.-X., Kalogerakis N. (2019). Biodegradation of mixture of plastic films by tailored marine consortia. J. Hazard Mater..

[18] Muthukumar T., Aravinthan A., Lakshmi K., Venkatesan R., Vedaprakash L., Doble M. (2011). Fouling and stability of polymers and composites in marine environment. Int. Biodeterior. Biodegrad..

[19] Sudhakar M., Priyadarshini C., Doble M., Sriyutha Murthy P., Venkatesan R. (2007). Marine bacteria mediated degradation of nylon 66 and 6. Int. Biodeterior. Biodegrad..

[20] Artham T., Doble M. (2009). Fouling and Degradation of Polycarbonate in Seawater: Field and Lab Studies. J. Polym. Environ..

[21] Artham T., Doble M. (2012). Bisphenol A and metabolites released by biodegradation of polycarbonate in seawater. Environ. Chem. Lett..

[22] Kasuya K.i., Takagi K.i., Ishiwatari S.i., Yoshida Y., Doi Y. (1998). Biodegradabilities of various aliphatic polyesters in natural waters. Polym. Degrad. Stabil..

[23] Papageorgiou G.Z., Bikiaris D.N., Achilias D.S., Papastergiadis E., Docoslis A. (2011). Crystallization and biodegradation of poly(butylene azelate): Comparison with poly(ethylene azelate) and poly(propylene azelate). Thermochim. Acta.

[24] Sridewi N., Bhubalan K., Sudesh K. (2006). Degradation of commercially important polyhydroxyalkanoates in tropical mangrove ecosystem. Polym. Degrad. Stabil..

[25] Tachibana K., Urano Y., Numata K. (2013). Biodegradability of nylon 4 film in a marine environment. Polym. Degrad. Stabil..

[26] Vaclavkova T., Ruzicka J., Julinova M., Vicha R., Koutny M. (2007). Novel aspects of symbiotic (polyvinyl alcohol) biodegradation. Appl. Microbiol. Biotechnol..

[27] Papageorgiou G.Z., Panayiotou C. (2011). Crystallization and melting of biodegradable poly(propylene suberate). Thermochim. Acta.

[28] Bikiaris D.N., Papageorgiou G.Z., Giliopoulos D.J., Stergiou C.A. (2008). Correlation between Chemical and Solid-State Structures and Enzymatic Hydrolysis in Novel Biodegradable Polyesters. The Case of Poly(propylene alkanedicarboxylate)s. Macromol. Biosci..

[29] Pan C., Lu J., Wu B., Wu L., Li B.-G. (2017). Effect of Monomer Structure on Crystallization and Glass Transition of Flexible Copolyesters. J. Polym. Environ..

[30] Pantani R., Sorrentino A. (2013). Influence of crystallinity on the biodegradation rate of injection-moulded poly(lactic acid) samples in controlled composting conditions. Polym. Degrad. Stabil..

[31] Mackintosh A.R., Liggat J.J. (2004). Dynamic mechanical analysis of poly(trimethylene terephthalate)—A comparison with poly(ethylene terephthalate) and poly(ethylene naphthalate). J. Appl. Polym. Sci..

[32] Turnbull L., Liggat J.J., MacDonald W.A. (2013). Thermal degradation chemistry of poly(ethylene naphthalate) – A study by thermal volatilisation analysis. Polym. Degrad. Stabil..

[33] Scheirs J., Gardette J.-L. (1997). Photo-oxidation and photolysis of poly(ethylene naphthalate). Polym. Degrad. Stabil..

[34] Liu B., Zhang J., Guo H. (2022). Research Progress of Polyvinyl Alcohol Water-Resistant Film Materials. Membranes.

[35] Bikiaris D.N., Karayannidis G.P. (1999). Effect of carboxylic end groups on thermooxidative stability of PET and PBT. Polym. Degrad. Stabil..

[36] Otsuka S., Kuwajima I., Hosoya J., Xu Y., Yamazaki M. (2011). 2011 International Conference on Emerging Intelligent Data and Web Technologies.

[37] Sahigara F., Ballabio D., Todeschini R., Consonni V. (2013). Defining a novel k-nearest neighbours approach to assess the applicability domain of a QSAR model for reliable predictions. J. Cheminf..

[38] Tokiwa Y., Calabia B.P., Ugwu C.U., Aiba S. (2009). Biodegradability of Plastics. Int. J. Mol. Sci..

[39] Seidi F., Zhong Y., Xiao H., Jin Y., Crespy D. (2022). Degradable polyprodrugs: design and therapeutic efficiency. Chem. Soc. Rev..

[40] Jablonka K.M., Patiny L., Smit B. (2022). Making the collective knowledge of chemistry open and machine actionable. Nat. Chem..

[41] Jaeger S., Fulle S., Turk S. (2018). Mol2vec: Unsupervised Machine Learning Approach with Chemical Intuition. J. Chem. Inf. Model..

