# Supplementary material for: Revealing factors influencing polymer degradation with rank-based machine learning
Source: Patterns (N Y). 2023 Sep 25;4(12):100846. doi: 10.1016/j.patter.2023.100846 (PMC10724228; doi:10.1016/j.patter.2023.100846)
Supplement: Document S1. Figures S1 and S2 [file mmc1.pdf]

Patterns, Volume *4*

Supplemental information

Revealing factors influencing polymer degradation with rank-based machine learning

Weilin Yuan, Yusuke Hibi, Ryo Tamura, Masato Sumita, Yasuyuki Nakamura, Masanobu Naito, and Koji Tsuda

Supplementary information

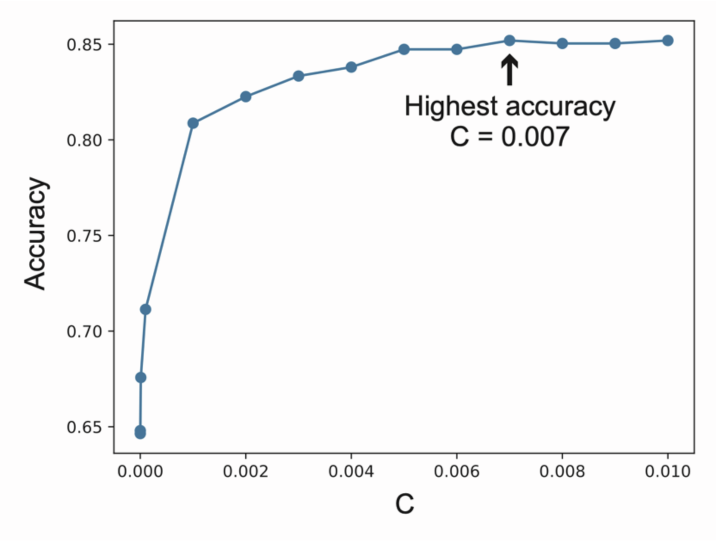

Figure S1. Accuracy for predictions of preferences depending on the value of C. The accuracy is evaluated by 5-fold cross-validation. When C = 0.007, the accuracy reaches its maximum value of 0.85.

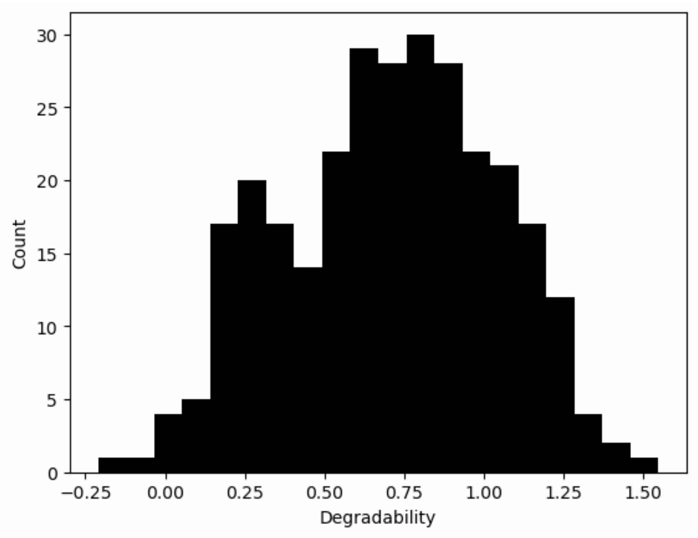

Figure S2. Predicted degradability for all polymers including isopropyl groups in PoLyInfo.
